# Pulmonary Metastasectomy for Head and Neck Squamous Cell Carcinoma in the Modern Treatment Era

**DOI:** 10.1093/icvts/ivag090

**Published:** 2026-03-26

**Authors:** Ryu Kanzaki, Hisaya Chikaraishi, Takuya Tokunaga, Hironobu Samejima, Masao Kobayashi, Tomohiro Maniwa, Takashi Fujii, Jiro Okami

**Affiliations:** Department of General Thoracic Surgery, Osaka International Cancer Institute, Osaka, 541-8567, Japan; Department of Head and Neck Surgery, Osaka International Cancer Institute, Osaka, 541-8567, Japan; Department of General Thoracic Surgery, Osaka International Cancer Institute, Osaka, 541-8567, Japan; Department of Head and Neck Surgery, Osaka International Cancer Institute, Osaka, 541-8567, Japan; Department of General Thoracic Surgery, Osaka International Cancer Institute, Osaka, 541-8567, Japan; Department of Head and Neck Surgery, Osaka International Cancer Institute, Osaka, 541-8567, Japan; Department of General Thoracic Surgery, Osaka International Cancer Institute, Osaka, 541-8567, Japan; Department of Head and Neck Surgery, Osaka International Cancer Institute, Osaka, 541-8567, Japan; Department of General Thoracic Surgery, Osaka International Cancer Institute, Osaka, 541-8567, Japan; Department of Head and Neck Surgery, Osaka International Cancer Institute, Osaka, 541-8567, Japan; Department of General Thoracic Surgery, Osaka International Cancer Institute, Osaka, 541-8567, Japan; Department of Head and Neck Surgery, Osaka International Cancer Institute, Osaka, 541-8567, Japan; Department of General Thoracic Surgery, Osaka International Cancer Institute, Osaka, 541-8567, Japan; Department of Head and Neck Surgery, Osaka International Cancer Institute, Osaka, 541-8567, Japan; Department of General Thoracic Surgery, Osaka International Cancer Institute, Osaka, 541-8567, Japan; Department of Head and Neck Surgery, Osaka International Cancer Institute, Osaka, 541-8567, Japan

**Keywords:** pulmonary metastasectomy, head and neck squamous cell carcinoma, immune checkpoint inhibitors, prognosis

## Abstract

**Objectives:**

Pulmonary metastasectomy (PM) has been a viable treatment option for metastatic head and neck squamous cell carcinoma (HNSCC). In recent years, advances in systemic therapy and multidisciplinary cancer management, including immune checkpoint inhibitors (ICIs), have changed the treatment landscape of advanced or recurrent head and neck cancer.

**Methods:**

We retrospectively reviewed 44 patients who underwent complete resection for pulmonary metastases from HNSCC between 2008 and 2021. To minimize the risk of including primary lung cancer, 8 patients were excluded based on predefined clinical criteria, resulting in a final cohort of 36 patients for analysis. Patients were divided into the early (2008-2016) and late (2017-2021) period groups. Relapse-free survival (RFS), overall survival (OS), and prognostic factors were assessed.

**Results:**

The 5-year RFS and OS for all patients were 41% and 67%, respectively. Postoperative chemotherapy was associated with better RFS, and age ≤65 years with better OS. ICIs were administered to 1 patient in the early period group and 7 in the late period group during the clinical course (*P* = .005). The 5-year RFS and OS were 42% vs 40% and 55% vs 87% in the early and late period groups, respectively. While RFS was comparable, OS tended to be better in the late period group, although the difference was not statistically significant.

**Conclusions:**

PM is associated with favourable long-term outcomes in patients with HNSCC. Given its potential for long-term survival and diagnostic value, PM remains an important treatment option for HNSCC in the modern treatment era.

## Graphical abstract

**Figure:**
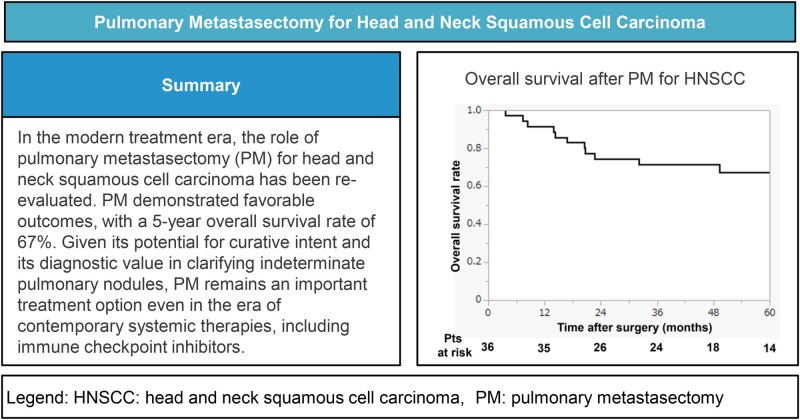

## INTRODUCTION

Head and neck cancers (HNCs) represent a significant global health burden, with head and neck squamous cell carcinoma (HNSCC) being the predominant histological type. In patients with HNSCC, the lungs are the most frequent site of distant metastasis, followed by bone and liver.^1^ While systemic therapy remains the standard treatment for recurrent or metastatic disease, long-term outcomes have been poor. Pulmonary metastasectomy (PM) has been performed in selected patients based on retrospective evidence suggesting a potential survival benefit.^2^ However, the absence of randomized controlled trials directly comparing surgery with systemic therapy has left its role open to discussion. Importantly, the role of PM has evolved alongside advances in systemic therapy.

In recent years, advances in systemic therapy and multidisciplinary cancer management, including the introduction of immune checkpoint inhibitors (ICIs), have significantly changed the treatment landscape of advanced HNSCC. Nivolumab and pembrolizumab were approved in Japan in 2017 and 2019, respectively, based on the CheckMate 141 and KEYNOTE-048 trials, which demonstrated improved overall survival (OS) in selected patient populations.^3^^,^^4^

In the modern treatment era, the clinical value of PM for lung metastases from HNSCC warrants re-evaluation, as reports incorporating contemporary systemic therapies, including ICIs, remain limited.^5^^,^^6^ Given the diagnostic difficulty in distinguishing pulmonary metastases from second primary lung cancer, predefined clinical exclusion criteria were used to reduce potential misclassification.^7^ This study evaluated clinical outcomes and prognostic factors of PM for lung metastases from HNSCC in the modern treatment era.

## PATIENTS AND METHODS

### Study design and ethical approval

The study protocol was approved by the Ethics Review Board of the Osaka International Cancer Institute (control number: 25016), and the requirement to obtain informed consent was waived. Between 2008 and 2021, 44 patients underwent complete resection of pulmonary metastases from HNSCC at the Osaka International Cancer Institute. Patients who underwent biopsy-only procedures or incomplete resection were excluded. In addition, patients in whom suspected metastatic pulmonary nodules remained unresected were classified as having macroscopic residual disease and were excluded from the analysis.

### Exclusion criteria for possible primary lung cancer

Due to the difficulty in distinguishing primary lung cancer from pulmonary metastases of HNSCC based solely on pathological findings, an exclusion analysis was conducted based on the criteria described by Kozu et al,^7^ who proposed clinical criteria for differentiating pulmonary metastases of oesophageal cancer from primary lung cancer. Cases meeting all of the following clinical criteria were considered as possible primary lung cancer and thus excluded from this analysis^7^: (1) disease-free interval (DFI) of ≥24 months, defined as the time from definitive treatment of the primary tumour to the initial detection of pulmonary nodules on follow-up imaging; (2) solitary pulmonary lesion; (3) no radiologic or clinical evidence of recurrence at the primary site after pulmonary resection. In our cohort, 8 patients met these criteria. After excluding these, 36 patients were included in the analysis in the present study. To explore outcomes across treatment periods in the modern era, which became available for HNSCC in Japan in 2017, patients were stratified into 2 cohorts: the early period group (2008-2016) and the late period group (2017-2021).

### Surgical indications and procedures

Pulmonary resection was considered in patients who met the following criteria when pulmonary metastases from HNSCC were suspected preoperatively: (1) complete resection of all pulmonary nodules was deemed feasible; (2) no mediastinal lymph node metastases were identified on preoperative imaging; (3) metastatic disease was confined to the lungs, or any extrapulmonary lesions were controlled or controllable; (4) locoregional control of the primary HNSCC had been achieved; (5) the patient had adequate performance status and pulmonary function to tolerate surgery. The extent of pulmonary resection was determined based on tumour characteristics and the patient’s general and pulmonary status. Sublobar resection was generally preferred for presumed metastatic disease if complete resection was achievable. When primary lung cancer was suspected, lobectomy with mediastinal lymph node dissection was typically performed. Sublobar resection or omission of lymph node dissection was considered in patients with limited physiological reserve. Intraoperative lavage cytology of the parenchymal margin was generally performed during sublobar resection, with additional resection for positive results, while omission or completion without further resection was allowed at the surgeon’s discretion when adequate macroscopic margins were achieved.^8^ Surgical approach and extent of resection were determined by tumour location and margin feasibility, with an open or thoracoscopic approach selected as appropriate; limited resection was preferred for peripheral lesions, whereas lobectomy was performed for central lesions.

### Perioperative evaluation and pathological diagnosis

Medical, surgical, and anaesthetic records were reviewed to evaluate operative details and postoperative complications. Complications were classified according to the Clavien-Dindo system,^9^ and complications were defined as grade ≥2 events. All surgical specimens were assessed by pathologists. When differentiation between primary lung squamous cell carcinoma and pulmonary metastasis from HNSCC was challenging, p16 immunohistochemistry was selectively performed.

### Postoperative management and follow-up

Postoperative chemotherapy was administered at the discretion of the treating head and neck surgeon. The use of postoperative chemotherapy after PM was dependent on the treatment era. In the earlier treatment period (until 2015), postoperative chemotherapy was occasionally administered when feasible, reflecting limited therapeutic options for recurrence after metastasectomy. From 2016 onward, postoperative chemotherapy was no longer routinely performed following an intentional institutional policy change, based on the lack of evidence supporting adjuvant treatment in this setting and the availability of effective systemic therapies including ICIs, at the time of recurrence. Patients were followed every 3-6 months, with surveillance including chest computed tomography (CT), physical examination, and laboratory testing. Follow-up data were collected through hospital records and communication with referring physicians. The follow-up period ranged from 3 to 180 months (median: 48 months).

### Statistical analysis

All statistical analyses were performed using JMP Pro version 17.0 (SAS Institute, Berkeley, CA, United States). Continuous variables were presented as mean ± standard deviation or median, as appropriate. OS and relapse-free survival (RFS) after pulmonary resection were estimated using the Kaplan-Meier method, and differences between the groups were assessed by the log-rank test. RFS was defined as the time from pulmonary resection to the first documented recurrence, death, or last follow-up. OS was defined as the time from surgery to death or last follow-up. Univariate Cox proportional hazards regression analyses were performed to evaluate prognostic factors. A *P*-value <.05 was considered statistically significant. All statistical tests were 2-tailed, and *P*-values were not adjusted for multiple comparisons.

## RESULTS

Patient characteristics are summarized in **Table 1**. The mean age of the 36 patients was 63.7 years, and most were male (86%). Twenty-one patients (58%) underwent PM in the early period (2008-2016) and 15 (42%) in the late period (2017-2021). The hypopharynx was the most common primary site (39%), and most patients had stage IV disease (70%). Overall patient characteristics were largely comparable between periods, except that permanent tracheostomy was significantly more frequent in the early period group (48% vs 0%, *P* = .002).

**Table 1.** Patient Characteristics According to Treatment Period

Factor	Early period (2008–2016) *N* = 21	Late period (2017–2021) *N* = 15	*P*-value
Age, mean ± SD	65.0 ± 7.3	62.1 ± 13.4	.402
Sex, M/F	17/4 (81/19%)	14/1 (93/7%)	.376
Primary site			.721
Hypopharynx	8 (38%)	6 (40%)	
Oropharynx	3 (14%)	3 (20%)	
Larynx	4 (19%)	0 (0%)	
Nasopharynx	2 (10%)	2 (13%)	
Tongue	2 (10%)	2 (13%)	
Nasal cavity	0 (0%)	1 (7%)	
Oral cavity	2 (10%)	0 (0%)	
Paranasal sinus	0 (0%)	1 (7%)	
Stage of primary tumour			.343
I	1 (5%)	1 (7%)	
III	5 (24%)	4 (27%)	
IVa	15 (71%)	8 (53%)	
IVb	0 (0%)	2 (13%)	
Treatment for primary tumour			.746
Chemoradiotherapy	10 (48%)	8 (53%)	
Surgery	6 (29%)	3 (20%)	
Surgery + chemoradiotherapy	1 (5%)	1 (7%)	
Surgery + radiotherapy	4 (19%)	2 (13%)	
Surgery + chemotherapy	0 (0%)	1 (7%)	
Brinkman index median (range)	927 ± 576	700 ± 556	.244
Body mass index < 18.5	6 (29%)	3 (20%)	.705
Permanent tracheostomy	10 (48%)	0 (0%)	.002
History of malignancy other than HNSCC	6 (29%)	8 (53%)	.175
Hypertension	5 (24%)	6 (40%)	.465
Diabetes mellitus	1 (5%)	1 (7%)	1.000
Hypothyroidism	1 (5%)	2 (13%)	.559
Arrhythmia	1 (5%)	1 (7%)	1.000
Renal dysfunction	3 (14%)	3 (20%)	.677

Abbreviation: HNSCC, head and neck squamous cell carcinoma; SD, standard deviation.

Factors associated with pulmonary resection are shown in **Table 2**. The median disease-free interval was 14 months. Most patients had unilateral (97%) and solitary (86%) pulmonary metastases. Video-assisted thoracic surgery (VATS) was used in 39%, and wedge resection was the most common procedure (58%). Mean tumour size on CT was 19 mm. Postoperative complications occurred in 31%, and 25% received postoperative chemotherapy. Postoperative chemotherapy consisted of S-1 (*n* = 6), cisplatin plus 5-fluorouracil (*n* = 3), and docetaxel, cisplatin, and 5-fluorouracil (*n* = 1). Among 26 patients who underwent wedge resection or segmentectomy, intraoperative lavage cytology was performed in 24, yielding negative results in 21. Of 3 patients with positive cytology, 2 achieved negative results after additional resection, and 1 was managed without further resection due to adequate macroscopic margins, with a negative margin confirmed on permanent pathology. Consequently, no patients had final microscopic residual disease. Postoperative complications (Clavien-Dindo grade ≥2) occurred in 11 patients (31%). Grade 2 complications included drug-induced haemolytic anaemia (*n* = 1), drug eruption (*n* = 1), pneumonia (*n* = 3), and hyponatremia (*n* = 1). Grade 3a complications consisted of atelectasis requiring bronchofiberscopic intervention (*n* = 2), prolonged air leak requiring chest drainage reinsertion or additional drainage (*n* = 2), and bronchopleural fistula treated bronchoscopically with fibrin glue (*n* = 1).

**Table 2.** Factors Associated With Pulmonary Resection According to Treatment Period

Factor	Early period (2008–2016) *N* = 21	Late period (2017–2021) *N* = 15	*P*-value
Disease-free interval			
Mean ± SD	15.5 ± 7.5	18.2 ± 12.4	.438
Median (range)	14 (4–37)	15 (6–49)	
Number of sides (unilateral/bilateral)	21/0 (100/0%)	14/1 (93/7%)	.417
Number or pulmonary tumors			.033
1	20 (95%)	11 (73%)	
2	0 (0%)	4 (27%)	
4	1 (5%)	0 (0%)	
Recurrence before pulmonary metastasis	6 (29%)	6 (40%)	.499
Chemotherapy before surgery for pulmonary metastasis	1 (5%)	2 (13%)	.559
CT tumour size			
Mean ± SD	23 ± 16	14 ± 8	.046
Median (range)	17 (8–80)	11 (6–35)	
Surgical approach, thoracotomy/VATS	16/5 (76/24%)	6/9 (40/60%)	.041
Type of pulmonary resection			.985
Lobectomy	6 (29%)	4 (27%)	
Segmentectomy	3 (14%)	2 (13%)	
Partial resection	12 (57%)	9 (60%)	
Operation time (minutes), mean ± SD	141 ± 81	125 ± 81	.579
Blood loss (g), mean ± SD	145 ± 226	27 ± 41	.052
Pathological tumour size			
Mean ± SD	22 ± 16	17 ± 4	.245
Median (range)	17 (8–80)	15 (6–45)	
Postoperative complications	8 (38%)	3 (20%)	.295

Abbreviation: CT, computed tomography; SD, standard deviation; VATS, video-assisted thoracic surgery.

**Figure 1.**
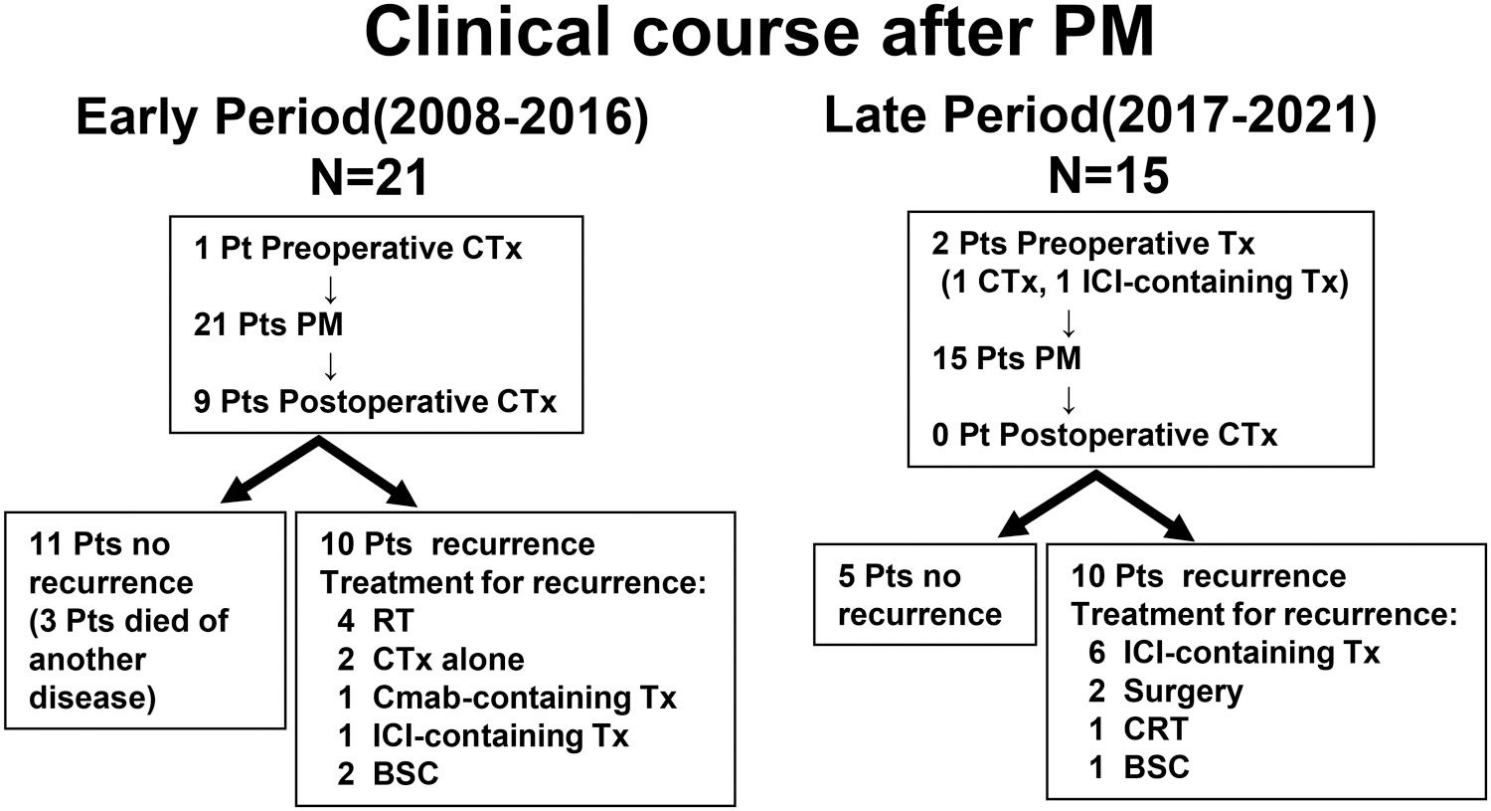
Clinical Course of Patients Undergoing Pulmonary Metastasectomy for Pulmonary Metastases From Head and Neck Squamous Cell Carcinoma. Patients were categorized into early (2008-2016) and late (2017-2021) periods. Abbreviations: BSC, best supportive care; Cmab, cetuximab; CRT, chemoradiotherapy; CTx, chemotherapy; ICI, immune checkpoint inhibitor; PM, pulmonary metastasectomy; RT, radiotherapy; Tx, therapy

**Figure 1** shows the clinical course of patients undergoing PM for pulmonary metastases from HNSCC according to treatment period. ICIs were used in 1 patient in the early period group and 7 patients in the late period group throughout the clinical course (*P* = .005). In all the 36 patients, the 5-year RFS and OS were 41% and 67%, respectively. Univariate analysis for factors influencing the RFS after pulmonary resection is shown in **Table 3**. Administration of postoperative chemotherapy was significantly associated with better RFS. Univariate analysis for factors influencing the OS after pulmonary resection is shown in **Table 4**. Age (≤65 years) was significantly associated with better OS.

**Table 3.** Univariate Analysis of Factors Influencing the Relapse-Free Survival

	Hazard ratio	95% CI	*P*-value
Age (>65/≤65)	2.04	0.86–4.85	.105
Sex (male/female)	2.1	0.49–9.00	.316
Treatment period (early/late)	1.11	0.48–2.61	.793
Primary site (pharynx/other)	0.71	0.31–1.65	.430
Primary stage (stage I-III/stage IV)	1.36	0.58–3.21	.484
Primary treatment (surgery inclusive/not)	1.84	0.79–4.26	.156
BMI (<18.5/≥18.5)	0.86	0.32–2.33	.773
Permanent tracheostomy (yes/no)	0.55	0.20–1.50	.241
History of malignancy other than HNSCC (yes/no)	1.06	0.46–2.42	.893
Brinkman Index (>600/≤600)	1.21	0.51–2.87	.668
DFI12 (>12 months/≤12 months)	1.16	0.49–2.73	.740
DFI24 (>24 months/≤24 months)	2.3	0.83–6.34	.107
Number of tumors (multiple/single)	1.28	0.43–3.82	.653
Recurrence before pulmonary metastasis (yes/no)	1.09	0.46–2.58	.843
Preoperative chemotherapy for pulmonary metastasis (yes/no)	0.88	0.21–3.79	.867
Tumour size (>2 cm/≤2 cm)	1.15	0.49–2.73	.748
Surgical approach (thoracotomy/VATS)	2.25	0.88–5.73	.089
Type of resection (lobectomy or segmentectomy/partial resection)	1.27	0.56–2.89	.566
Postoperative complications (yes/no)	0.94	0.38–2.28	.887
Postoperative chemotherapy (yes/no)	0.29	0.09–0.99	.048

Abbreviations: BMI, body mass index; CI, confidence interval; DFI, disease-free interval; VATS, video-assisted thoracic surgery.

**Table 4.** Univariate Analysis of Factors Influencing the Overall Survival

	Hazard ratio	95% CI	*P*-value
Age (>65/≤65)	3.69	1.02–13.30	.046
Sex (male/female)	0.97	0.21–4.39	.966
Treatment period (early/late)	2.47	0.66–9.17	.178
Primary site (pharynx/other)	0.5	0.17–1.45	.204
Primary stage (stage I-III/stage IV)	1.52	0.50–4.57	.457
Primary treatment (surgery inclusive/not)	2.02	0.68–6.06	.208
BMI (<18.5/≥18.5)	2.42	0.78–7.46	.124
Permanent tracheostomy (yes/no)	0.55	0.20–1.50	.241
History of malignancy other than HNSCC (yes/no)	1.1	0.38–3.16	.865
Brinkman Index (>600/≤600)	2.4	0.67–8.63	.179
DFI12 (>12 months/≤12 months)	0.73	0.25–2.10	.557
DFI24 (>24 months/≤24 months)	0.93	0.21–4.17	.923
Number of tumors (multiple/single)	0.4	0.05–3.05	.375
Recurrence before pulmonary metastasis (yes/no)	0.57	0.16–2.06	.393
Preoperative chemotherapy for pulmonary metastasis (yes/no)	1.79	0.39–8.19	.454
Tumour size (>2 cm/≤2 cm)	1.66	0.57–4.82	.350
Surgical approach (thoracotomy/VATS)	2.26	0.53–8.17	.214
Type of resection (lobectomy or segmentectomy/partial resection)	0.91	0.31–2.62	.854
Postoperative complications (yes/no)	1	0.31–3.19	.998
Postoperative chemotherapy (yes/no)	0.56	0.15–2.10	.391

Abbreviations: BMI, body mass index; CI, confidence interval; DFI, disease-free interval; VATS, video-assisted thoracic surgery.

**Figure 2.**
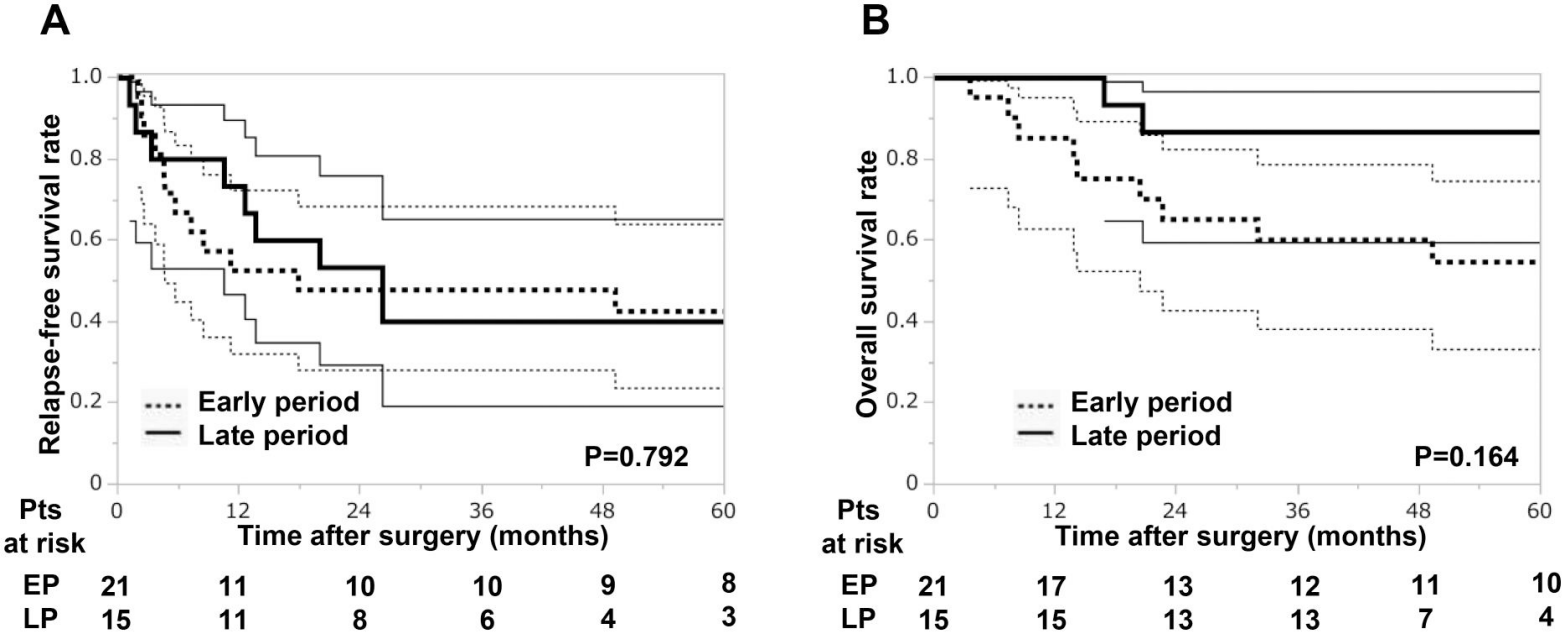
Long-Term Outcomes After Pulmonary Resection for Pulmonary Metastases From Head and Neck Squamous Cell Carcinoma by Treatment Period. (A) Relapse-free survival (RFS). (B) Overall survival (OS). Five-year RFS and OS were comparable between periods for RFS but tended to be higher in the late period for OS (*P* = .164). Bold lines indicate Kaplan-Meier estimates and thin lines the 95% confidence intervals

Next, we compared long-term outcomes between the early (2008-2016) and late (2017-2021) period groups. The 5-year RFS and OS were 42% (95% CI 21%-64%) vs 40% (95% CI 15%-65%) and 55% (95% CI 33%-77%) vs 87% (95% CI 69%-100%) in the early and late period groups, respectively. While RFS was comparable between the groups, OS tended to be better in the late period group, although the difference did not reach statistical significance (*P* = .164) (**Figure 2**).

## DISCUSSION

PM for lung metastases from HNSCC was associated with favourable long-term survival. Improvements in OS over time likely reflect advances in systemic therapy and multidisciplinary care, including ICIs, supporting periodic re-evaluation of the role of PM as treatment strategies evolve.

In the present study, to address the diagnostic difficulty in distinguishing solitary pulmonary metastases from HNSCC from primary lung cancer, we applied strict exclusion criteria reported by Kozu et al^7^; even after this adjustment, the 5-year OS rate remained 67%. Our findings are consistent with prior studies supporting the role of PM in carefully selected patients. Shiono et al reported 5-year OS rates of 20%-63% following PM.^5^^,^^10–19^ In our cohort, the 5-year OS rate in the late period group (2017-2021), within the ICI era, was 87%, compared to 55% in the early period group (2008-2016). Although not statistically significant, this improvement may reflect recent advances in diagnostic imaging and systemic therapy, including the increasing use of ICIs.

In addition to advances in systemic therapy, several clinicopathological factors differed between the early and late periods and may have influenced outcomes. In the late period, PM tended to be detected at a smaller size, whereas the proportion of patients with multiple pulmonary lesions was higher. From an oncological perspective, smaller tumour size may favour improved prognosis, while multiple lesions are generally associated with poorer outcomes.^2^ These opposing trends suggest that the overall oncological burden was not uniformly reduced in the late period group. VATS adoption increased over time with technical advances, while surgical indications and oncological principles remained unchanged, achieving adequate margins with reduced invasiveness. Collectively, these findings indicate that multiple factors beyond ICI use alone may have contributed to outcome differences between the early and late periods.

Recent studies on PM outcomes in HNC in the modern treatment era (since 2017) are summarized in **Table 5**.^5^^,^^10–19^ Across 9 retrospective studies, 5-year OS ranged from 36% to 59% for squamous cell carcinoma. Only 2 previous reports explicitly stated how many patients received ICIs during the whole clinical course: report by Schlachtenberger et al (*n* = 11) and Ochi et al (*n* = 4).^5^^,^^6^ Incomplete resection, larger size, and shorter DFI consistently correlated with worse prognosis. One large cohort reported a 5-year OS of 53% in the surgery group vs 20% in the non-surgical group.^19^ Our cohort achieved a 67% 5-year OS rate, likely reflecting the high proportion of solitary pulmonary lesions (86% in our cohort vs 52%-87 % in earlier series). These findings suggest that careful selection of patients with HNSCC pulmonary metastases can yield excellent long-term outcomes after PM. It was also suggested that postoperative chemotherapy may have contributed to delaying recurrence in the present study. The observed difference in postoperative chemotherapy between periods reflects a deliberate institutional policy change rather than selection bias. With the advent of ICIs, the clinical strategy shifted towards reserving chemotherapy for recurrence, where meaningful therapeutic benefit could be expected. Collectively, these results underscore the importance of individualized patient selection and integration of perioperative systemic therapy when considering PM in the modern treatment era.

**Table 5.** Summary of Recent Studies on Pulmonary Metastasectomy Outcomes in Head and Neck Cancer in the Modern Treatment Era (Since 2017)

Year	Author	No. of patients	No. of pulmonary lesion(s) (solitary/multiple)	Survival after PM	Worse prognostic factors after PM
2017	Nakajima^11^	58	43/15	5-year OS 36%	DFI < 24 months, oral-cavity primary
2019	Oki^14^	77	67/10	5-year OS 54%	SCC histology, shorter DFI, recurrence before pulmonary metastasis, larger size
2019	Lu^13^	46	24/22	2-year OS: SCC 59%	SCC histology, a time to distant metastasis after curative treatment of ≤12 months
2019	AlShammari^15^	56	N.A.	5-year OS: SCC 45%	SCC histology
2021	Dudek^16^	44	N.A.	5-year OS 41%	Larger size
2021	Ochi^5^	25	15/10	5-year OS: SCC 43%	Local recurrence in the primary site before pulmonary metastasis
2022	Kuroda^17^	231	181/50	5-year OS 43%	Oral/tongue/sinus primary; larger size
2022	Park^18^	54	N.A.	5year DFS: SCC 40%	DFI < 14 months, R1 resection
2022	Schlachtenberger^19^	33	N.A.	5-year OS 53%	N.A.
	This study	36	31/5	5-year OS 67%	No postoperative chemotherapy after PM for RFS, age <65 years for OS

Abbreviations: DFI, disease-free interval; HNSCC, head and neck squamous cell carcinoma; MST, median survival time; N.A., not available; PM, pulmonary metastasectomy; RFS, relapse-free survival; OS, overall survival; SCC, squamous cell carcinoma.

A persistent clinical challenge in this setting is the distinction between pulmonary metastasis from HNSCC and second primary lung cancer, particularly given their overlapping squamous histology. A previous study reported that among 39 HNSCC patients undergoing pulmonary resection, 38% had pulmonary metastases, whereas 62% were ultimately diagnosed with primary lung cancer.^20^ Histopathological evaluation alone is often inconclusive because of substantial morphological similarity. To aid in this differentiation, Ichinose et al proposed an immunohistochemistry-based algorithm using CK19, MMP3, and PI3, which demonstrated high sensitivity with moderate diagnostic accuracy in distinguishing pulmonary metastases of HNSCC from primary lung squamous cell carcinoma.^21^ At our institution, p16 immunohistochemistry was selectively performed as part of routine clinical practice, and strong, diffuse p16 positivity was interpreted as suggestive of HPV-related oropharyngeal metastasis rather than primary pulmonary carcinoma in appropriate clinical contexts. Nevertheless, despite the use of predefined clinical exclusion criteria and adjunctive immunohistochemical evaluation, definitive discrimination remains challenging in some cases. This diagnostic uncertainty is inherent to real-world clinical practice and should be considered when interpreting survival outcomes after PM.

For solitary pulmonary nodules of uncertain origin, surgical resection continues to offer both therapeutic benefit and diagnostic clarification, especially in smoking-related cancers where the risk of second primary lung cancer is elevated. On the other hand, for patients unfit for surgery due to advanced age, comorbidities, or poor pulmonary function, stereotactic body radiation therapy (SBRT) provides a viable alternative. It is demonstrated that SBRT offers comparable 3-year local control to PM (92% vs 88%) with similar complication rates, supporting its use in high-risk patients.^22^ In patients with a history of HNC who develop pulmonary nodules with a high suspicion of malignancy, it is prudent to select either surgery or SBRT according to the patient’s overall clinical condition.

This study has several limitations. As a retrospective analysis conducted over a decade, changes in diagnostic standards, systemic treatments, and surgical selection criteria may have influenced the outcomes. Distinguishing pulmonary metastases from second primary lung cancer remains challenging and may have introduced misclassification bias affecting survival analyses. Although predefined criteria and multidisciplinary assessment were applied, residual diagnostic uncertainty cannot be excluded.

## CONCLUSIONS

PM is associated with favourable long-term outcomes in patients with HNSCC. In the modern treatment era—characterized by advances in systemic therapy, including ICIs—PM remains an important therapeutic and diagnostic option in carefully selected patients.

## Data Availability

The datasets used and/or analysed during the current study are available from the corresponding author upon reasonable request.
